# Prevalence of modifiable risk factors of tuberculosis and their population attributable fraction in Iran: A cross-sectional study

**DOI:** 10.1371/journal.pone.0271511

**Published:** 2022-08-04

**Authors:** Kamal Sadeghi, Jalal Poorolajal, Amin Doosti-Irani

**Affiliations:** 1 Department of Epidemiology, School of Public Health, Hamadan University of Medical Sciences, Hamadan, Iran; 2 Modeling of Noncommunicable Diseases Research Center, Hamadan University of Medical Sciences, Hamadan, Iran; 3 Research Center for Health Sciences, Hamadan University of Medical Sciences, Hamadan, Iran; Tabriz University of Medical Sciences, ISLAMIC REPUBLIC OF IRAN

## Abstract

**Background:**

The mycobacterium tuberculosis (Mtb) is necessary for the morbidity of tuberculosis (TB), but it is insufficient. Many risk factors increase the risk of disease among infected people. We aimed to estimate the prevalence of modifiable risk factors of TB and their related population attributable fraction (PAF) in the marginal population of Markazi province in Iran.

**Design:**

In this cross-sectional study, the prevalence of the modifiable risk factors of TB was estimated. We designed and validated a questionnaire to determine the risk factors. The measures of association for the modifiable risk factors of TB were obtained via the review of published literature. We calculated the PAF for each modifiable risk factor.

**Results:**

Out of the 1275 calculated sample size, 1146 people participated in this study, and the participation rate was 89.9%. The mean age was 39.26. Out of 1146 participants, 76% did not know anything regarding TB. The highest prevalence was related to the lack of physical activity (58.73%), lack of fish consumption (50.79%), lack of red meat consumption (21.20%), and secondhand smoke (19.02%). The highest PAF was related to secondhand smoke; this value based on the crude relative risk (RR) and crude odds ratio (OR) was 24.54% and 23.44%, respectively. Based on crude hazard ratio (HR) and crude OR, the PAF for smoking was 14.81% and 11.19%, respectively. PAF for lack of BCG vaccination based on the crude OR was 14.79%.

**Conclusion:**

Based on this study’s results, poor nutrition, secondhand smoke, smoking, lack of BCG vaccination, and diabetes are the main prevalent modifiable risk factors for TB. The highest PAF for TB was related to secondhand smoke, smoking, lack of BCG vaccination, and diabetes.

## Introduction

Tuberculosis (TB), as a significant infectious disease, is one of the top ten causes of mortality in the world [1]. In 2019 about 10 million people were affected by TB globally; this year, about 1.2 million deaths occurred among HIV-negative people, and an additional 208000 deaths occurred among HIV-negative people. About 8.2% of all TB patients in 2019 were reported in the Eastern Mediterranean region of the world health organization (WHO) [1]. The total new cases and incidence rate of TB in Iran in 2019 were 11000 (8200, 13000) and 13.0 (9.8, 16.0) pre 100, 000 respectively [2].

Although TB is an infectious disease and mycobacterium tuberculosis (Mtb) is necessary to the morbidity of the disease, it is not sufficient. Many risk factors, such as age, HIV infection, smoking, dwelling density, body mass index, alcohol consumption, low socioeconomic status, working environment for health care workers, lack of optimum knowledge, attitude, and practice regarding preventive measures of TB, illiteracy, and having a household member with TB increase the risk of the disease [3–6]. Some of these risk factors are modifiable, and reducing these factors may be helpful in the prevention of TB. Some studies estimated the prevalence of tuberculosis and evaluated the association of modifiable risk factors of TB [5, 7]. The attributable population fraction (PAF) is the fraction of all disease cases in a population attributed to a specific risk factor. PAF as an epidemiologic measure is applicable to assess the public health impact of exposures in people [8]. Calculation of PAF for modifiable risk factors of TB, as a multifactorial disease, can be helpful for public health interventions.

Some studies estimated the population attributable fraction (PAF) for some of the risk factors of TB. PAF for living in a rural area, lower than average family annual income per capita, history of TB, underweight, diabetes, and close contacts with TB patients was 50.67%, 30.42%, 12.70%, 5.52%, 50.50%, and 1.70% respectively [7].

But it seems evidence regarding the prevalence of modifiable risk factors of TB and their related PAF is insufficient. Unbiased estimation of the population attributable risk for modifiable risk factors of TB can be helpful for health policymakers in sound planning for primary prevention activities [9, 10].

According to WHO’s End TB Strategy, the percentage reduction in the incidence rate of TB per 100 000 population in 2025, 2030, and 2035 compared to 2015 is 50%, 80%, and 90%, respectively [1]. To achieve these goals, it seems in addition to case findings, systematic screening of contacts and high-risk groups, treatment of patients, management of comorbidities of TB/HIV, and preventive treatment for high-risk populations [11], we should think of the primary prevention.

Appropriate planning for primary prevention of TB requires knowledge regarding the prevalence of modifiable risk factors of TB and their PAF. Therefore, in this study, we aimed to estimate the prevalence of modifiable risk factors of TB and their related PAF in the marginal population of Markazi province.

## Methods

### Study design and setting

The ethics committee of Hamadan University of Medical Sciences approved the proposal of this study (IR.UMSHA.REC.1398.818). In addition, written informed consent was obtained from all participants. We designed a cross-sectional survey in Markazi province in 2020 to estimate the prevalence of the risk factors of TB.

In the first step, we review published systematic reviews and observational studies to identify the modifiable risk factors of TB. The major international databases, including Web of Sciences, PubMed, and Scopus, were searched using the following search strategy: #1 Tuberculosis, # 2 TB, #3: (#1 OR #2); #4: Risk factors [Mesh term], #5: Case-control studies [Mesh term], #6 case based studies [tw], #7 "case-reference studies" [tw], #8 Cohort Studies [Mesh term], #9 Longitudinal Studies [Mesh term], #10 Systematic Review [Publication Type], #11 Systematic Reviews as Topic [Mesh term], #12 Meta-Analysis [Publication Type], #13 Meta-Analysis as Topic [Mesh term]; # 14 (#5 OR #6 OR #7 OR #8 OR #9 OR #10 OR #11 OR #12 OR #13); #15 (#3 AND #4 AND #14).

The eligibility criteria for including studies were the analytic observational studies that assess the association of the risk factors of TB, regardless of the location of study, time, and language of publication.

Two investigators (KS, ADI) screened the retrieved studies. After removing the duplicated studies, the mentioned investigators screened the studies based on title and abstract, any disagreement between them was resolved by discussion. The full text of included studies was assessed based on the mentioned eligibility criteria in the next step. Finally, the following data were extracted and imported into a datasheet: name of the first author, year of publication, location, study population, study design, sample size, type of risk factors, and the measures of association with TB, including relative risk (RR), hazard ratio (HR), and odds ratio (OR) with their 95% confidence interval (CI). Two investigators (KS, ADI) assessed the quality of included studies using the Newcastle-Ottawa Scale for observational studies [12]. In the cases, we found more than one study regarding the association between modifiable risk factors and TB; we pooled the measures of association using the random-effects meta-analysis (S1 and S2 Tables in S1 File).

### Participants

In this cross-sectional study, the eligibility criteria for participation were living on the edge of towns with more than 40,000 populations in Markazi province and adults more than 18 years old. Four cities in Markazi province, including Arak, Khomain, Mahalat, and Saveh, were selected for this study. The sampling approach in this study was simple random sampling and was proportion to the size of each city. We calculated the sample size based on the results of our pilot study. In the pilot study, the prevalence of secondhand smoking (p) as a risk factor for TB was 6.67%. The alpha value was considered 0.05, and d was considered 0.20×p. The following formula was used for sample size calculation.


$$n=\frac{Z_{1-\alpha /2}^{2}\times p(1-p)}{d^{2}}$$

Finally, we reached to 1275 sample size for this study. The assigned samples in Arak, Khomain, Mahalat, and Saveh were 956, 128, 38, and 153.

### Measurements

We designed a questionnaire to determine the prevalence of modifiable risk factors of TB. Based on the literature review in the previous step, we extracted the related items and selected 30 risk factors. The criteria for the selection of risk factors was modifiability of them. We did not include the unmodifiable risk factors. In the next step, ten experts in TB, infectious diseases, and epidemiology were invited to determine the content validity of the questions. Six experts accepted our invitation and evaluated the content validity of the questionnaire. The scale content validity index for necessity, relevancy, clarity, and simplicity was 74.4%, 82.8%, 88.9%, and 92.8%. The reliability of the questionnaire was evaluated in a pilot study with 30 participants. The test-retest approach was used to determine the reliability. The kappa statistic was calculated to assess the agreement between the responses in the test and retest stages. The final questionnaire involved questions in three sections. The questions in the first section were related to participants’ baseline and demographic characteristics. Questions in the second section were associated with chronic diseases, including diabetes, hypertension, kidney diseases, chronic respiratory diseases, other diseases, and history of organ transplantation. The third section was related to the knowledge regarding TB. The fourth section of the questionnaire was associated with the modifiable risk factors of TB.

A researcher-designed questionnaire evaluated the socioeconomic status of participants. This questionnaire was based on people’s assets. The used assets include a freezer, washing machine, LCD/LED TV, microwave, vacuum cleaner, smartphone, dishwasher, and personal care (Not to earn money). We calculated the Wealth index using the principal component analysis (PCA) [13]. According to the PCA results, participants were classified into five groups from the lowest (quintile 1) to the highest (quintile5) level of socioeconomic status (SES).

### Statistical analysis

We calculated the population attribution fraction (PAF) for each modifiable risk. PAF was calculated indirectly by Levin’s equation [14]. This measure is defined as the fraction of the disease risk in the population associated with exposure [14]. PAF is an epidemiologic measure to assess the public health impact of risk factors in the populations and is a valuable measure to evaluate the effect of a public health intervention on reducing the risk factors [8, 15]

In this study, we used the crude estimates of OR, RR, and HR to calculate PAF because the assumption for the use of Levin’s equation is not confounding. Observational studies are not free of confounding, so it is inappropriate to use the adjusted measure of association to calculate the adjusted PAF [8, 14].


$$PAF=\frac{P_{e}\times (RR-1)}{P_{e}\times (RR-1)+1}\times 100$$

*Pe* is the risk factor prevalence, and RR is crude relative risk. Two critical elements of Levin’s equation are relative risk and prevalence of exposure [14]. Based on the results of our literature review, we have three types of measure of association, including crude OR, crude RR, and crude HR; therefore, in the above formula, in the cases of OR and HR, we replaced them with RR [16]. We could not use Miettinen’s equation to calculate because we have not accessed the prevalence of modifiable risk factors in TB patients. Based on the literature review, we obtained the crude measure of association for seven risk factors, including lack of BCG vaccination, closed contact with a TB patient, secondhand smoking, smoking, alcohol consumption, diabetes, and being underweight. Therefore, we estimated the PAF for these risk factors.

The continuous variables were reported as mean and standard deviation (SD), and categorical variables as percent and frequency. Prevalence of the modifiable risk factors for TB was reported with a 95% confidence interval (CI). Stata 14.2 (StataCorp, TX, US) was used for data analysis.

## Results

### Measures of association for modifiable risk factors of TB

In the first step of the study, out of 116 retrieved studies, 18 studies met the eligibility criteria and were included in the study (S1 Fig). The included studies evaluated the association of the following risk factors with TB: alcohol consumption, lack of BCG scar, underweight, close contact with a TB patient, family history of TB, diabetes, opium use, smoking, and secondhand smoke (S1 Table in S1 File). Based on the results of our literature review, the most robust measures of association for TB were related to the closed contact with a TB patient (crude OR: 2.83 (0.49, 5.17)), secondhand smoke (crude RR: 2.71 (1.01, 7.28), crude OR: 2.61 (1.08, 6.28)), and smoking (crude HR: 2.27 (1.36, 3.78)) respectively. Results of pooled measure of associations for the risk factors of TB are shown in S2 Table in S1 File.

### Prevalence of modifiable risk factors of TB

Out of the 1275 calculated sample size, 1146 people participated in this study, and the response rate was 89.9%. The mean and standard deviation (SD) for age were 39.26 and 13.4, respectively. Forty-four and seven percent of participants were male. In terms of socioeconomic status (SES), 28.36% and 20.07% of participants were in the poor (Quintile 2) and very poor (Quintile 1) status, respectively (Table 1). Out of 1146 participants, 76% did not know anything regarding TB. Of participants who had known the TB (275 participants), 80.73% knew the correct transmission route of TB, and 7.27% considered themselves at the risk of TB (Table 2).

**Table 1:** Baseline characteristics of participants.

**Continues variables**	**Mean**	**SD**
Age (yr)	39.26	13.41
Body mass index	26.23	4.30
Family size	3.59	1.15
Residential house area	98.71	31.84
Living rooms	1.38	0.58
The area of each room	10.15	3.42
**Categorical variables**	**Frequency**	**Percent**
**Gender**		
Female	634	55.32
Male	512	44.68
**Age group** (yr)		
<30	326	28.45
30–60	730	63.70
>60	90	7.85
**Nationality**		
Iranian	1127	98.34
Not Iranian	19	1.66
**Education**		
Illiterate	114	9.95
Elementary	224	19.55
Intermediate	248	21.64
Diploma	355	30.98
Associate degree	90	7.85
Bachelor	98	8.55
Master degree and upper	17	1.48
**Marital status**		
Single	156	13.61
Married	930	81.15
Divorced	21	1.83
Widow	39	3.40
**History of chronic diseases**		
Diabetes	120	10.47
Hypertension	170	14.83
Kidney disease	34	2.97
Respiratory diseases	14	1.22
Other diseases	63	5.50
**Socioeconomic status**		
Quintile 1	230	20.07
Quintile 2	325	28.36
Quintile 3	138	12.04
Quintile 4	225	19.63
Quintile 5	228	19.90

**Table 2:** The knowledge of participants regarding TB.

Variables	Frequency	Percent
**Knowledge regarding TB**		
Nothing about tuberculosis	871	76.00
**Transmission routes of TB**		
Respiratory	222	80.73
Oral	10	3.64
Injection	2	0.73
I don’t know	41	14.19
**Increase risk of TB with living with a people with TB**		
Yes	199	72.36
No	49	17.82
I don’t know	27	9.82
**TB is a treatable disease**		
Yes	214	77.82
No	29	10.55
I don’t know	32	11.64
**Do you consider yourself at risk for TB?**		
Yes	20	7.27
No	255	92.73

The highest prevalence was related to the lack of physical activity (58.73%), lack of fish consumption (50.79%), lack of red meat consumption (21.20%), and secondhand smoke (19.02%). The lowest prevalence was related to the history of contact with a TB patient (1.66%) and alcohol consumption (2.44%). The prevalence of other modifiable risk factors for TB is shown in Table 3.

**Table 3:** Prevalence of modifiable risk factors for TB.

Variables	Frequency	Percent
Lack of BCG vaccination	191	16.67
Closed contact with a TB patient	19	1.66
Working in a wet or dark place	82	7.16
Use of fossil fuels	57	4.97
Corticosteroid use history	17	1.48
Lack of sufficient sunlight in the house	133	11.61
Lack of physical activity	673	**58.73**
Underweight	17	1.48
Lack of red meat consumption in the last two weeks	243	**21.20**
Lack of chicken consumption in the last two weeks	90	7.85
Lack of fish consumption in the last two weeks	582	**50.79**
Lack of consumption of legumes in the last two weeks	64	5.58
Jail history	14	1.22
Jail history in family members	23	2.01
Smoking	157	13.70
Secondhand smoke	218	**19.02**
Alcohol consumption	28	2.44
Drug use	15	1.31

### PAF for modifiable risk factors of TB

The PAF was calculated for seven risk factors: lack of BCG vaccination, close contact with a TB patient, smoking, secondhand smoke, alcohol consumption, diabetes, and being underweight. The highest PAF was related to secondhand smoke. Based on the crude relative risk (RR) and crude odds ratio (OR), this value was 24.54% and 23.44%, respectively. Based on crude hazard ratio (HR) and crude OR, the PAF for smoking was 14.81% and 11.19%, respectively. PAF for lack of BCG vaccination based on the crude OR was 14.79%. The lowest PAF was related to alcohol consumption and being underweight (Table 4).

**Table 4:** Prevalence and population attributable fraction (PAF) for modifiable risk factors of TB.

Risk factors	Prevalence (95% CI)	Type measure of association	Measure of association	PAF (%)
Secondhand smoke	19.02 (16.85, 21.40)	Crude RR	2.71 (1.01, 7.28)	24.54
		Crude OR	2.61 (1.08, 6.28)	23.44
Smoking	13.70 (11.83, 15.82)	Crude HR	2.27 (1.36, 3.78)	14.81
		Crude OR	1.92 (1.23, 2.62)	11.19
Lack of BCG vaccination	16.67 (14.61, 18.94)	Crude OR	2.04 (1.32, 2.75)	14.77
Diabetes	10.47 (8.82, 12.38)	Crude RR	2.03 (1.62, 2.55)	9.73
		Crude OR	1.26 (0.62, 1.90)	2.65
Closed contact with a TB patient	1.66 (1.06, 2.59)	Crude OR	2.83 (0.49, 5.17)	2.93
Being underweight	1.48 (0.09, 2.37)	Crude OR	2.01 (1.36, 2.66)	1.47
Alcohol consumption	2.44 (1.69, 3.52)	Crude OR	1.67 (1.05, 2.28)	1.61
		Crude HR	1.45 (1.11, 1.90)	1.09

## Discussion

Based on the results of this study, a considerable proportion of participants have not known anything regarding TB. Among the specific risk factors of TB, lack of BCG vaccination, lack of sufficient sunlight in the house, and working in a wet or dark place have more prevalent. The prevalence of contact with a TB patient, use of fossil fuels, and corticosteroid use history were lower than other risk factors. Lack of red meat, fish, chicken, and legumes consumption in the last two weeks as predisposing factors of TB was considerable among participants. In addition, the prevalence of smoking and exposure to secondhand smoke was high among participants. The prevalence of jail history, alcohol consumption, and drug use were lower than other risk factors. The highest PAF was related to secondhand smoking, smoking, and lack of BCG vaccination.

Although TB is an infectious disease and mycobacterium tuberculosis (Mtb) is necessary for morbidity, it is insufficient. About 10 percent of people infected with Mtb progress to active TB during their lifetime. The reminded proportion of infected people have a latent state of the infection, and the pathogen persists for many years and can be reactivated and lead to active TB [17]. Factors such as the absence of BCG, crowding, HIV/AIDS, poor nutrition, and poor ventilation can increase active TB risk among people exposed to Mtb and individuals with latent TB.

Based on our results, 16.67% of participants did not have BCG vaccination, and the PAF for this risk factor was 14.77%. The high prevalence of lack of BCG vaccination and its PAF in our study is due to the high proportion of middle-aged and older adults among participants. The coverage of BCG vaccination among middle-aged and older adults may be lower than younger. Based on the national immunization program in Iran, all infants at birth receive the BCG vaccine [18]. Fortunately, the coverage of BCG vaccination in the recent decades is about 100% [19], so we expected the PAF for lack of BCG vaccination would be low for younger people in Iran. Our results regarding the BCG vaccination emphasize the importance of BCG vaccination.

Close contact with a TB patient is a strong risk factor for TB [15–18]; however, because of the low prevalence of this risk factor, the calculated PAF was low (2.93%). A reason is a relatively lower incidence of TB in Iran. Based to WHO, the total TB incidence rate in Iran in 2020 was 13.0 (95% CI: 9.6, 16.0) per 100,000 population [2]. According to a population-based cross-sectional study in China, the PAF based on the adjusted OR for closed contact was 1.71% [7], which is lower than our estimate; in this study, the authors used the Levin formula for calculating PAF. However, The Levin formula is valid for an unadjusted measure of association such as RR, OR, and HR [10, 14].

Smoking is another modifiable risk factor with a strong association with TB, and its prevalence in our study is about 14%. Based on our pooled crude HR and OR, the PAF for smoking was 14.81% and 11.19%, respectively. The prevalence of secondhand smoking was high, and the highest value of PAF was related to this risk factor. The prevalence of smoking among TB patients is more than in the general population; based on the results of the study prevalence of smoking in patients with pulmonary TB in Bangladesh and Pakistan was 28.3% and 22.0%, respectively [20, 21], on the other hand, smoking in TB patients raises the risk of infection in their relations. It is estimated that 12.8% of infections are due to smoking [22]. Therefore, reducing the prevalence of secondhand smoking, especially among patients with TB, can play an essential role in lowering TB infection in communities.

The prevalence of diabetes in our study was 10.47%, and its association with TB was strong based on the crude OR and crude RR. The PAF for diabetes based on crude RR was 9.73%. This finding does not align with a Chines study [7], which reported this index as 5.50% based on adjusted OR. In addition, the prevalence of diabetes in our study (10.47%) is more than in the mentioned study (7.01). Therefore, a reason for more PAF in our study than Chinses is the higher prevalence of diabetes and the robust measure of association in our study. PAF is affected by both measures of association and prevalence of exposure [14]. There is innate immune dysfunction among diabetic patients and susceptibility to TB. The function of the components of innate immunity, such as neutrophils, macrophages, DC, and NK cells, is severely affected by metabolic alterations in patients with diabetes. Therefore, immune dysfunction may have a significant role in the reactivation of TB infection and susceptibility of the host [23]. So the control of diabetes in the communities maybe have an essential role in preventing TB.

Our estimate for the prevalence of alcohol consumption was 2.44%, and the relative PAF was 1.61% and 1.09% based on the crude OR and crude RR, respectively. Alcohol consumption is a sensitive issue in Iran, so it is expected we underestimated the prevalence of this risk factor and, consequently, its PAF. A systematic review and meta-analysis showed that the last 12-month alcohol consumption prevalence varied from 0.03% to 68%, and the pooled estimates for the general population and young people were 12.0% (95% CI 7.0, 18.0) and 15.0% (95% CI 9.0, 22.0) respectively [24]. Therefore, the estimated PAF for alcohol consumption should be interpreted more cautiously.

The prevalence of drug use was 1.31%. We had no access to a crude measure of association for calculating the PAF for drug use. Like alcohol consumption, drug use is a sensitive issue, so individuals usually do not want to answer the sensitive questions correctly. The evaluation of the risk factors in our study was based on the self-declaration of participants. In the sensitive issues such as alcohol consumption and drug use, results are affected by reporting bias and consequently underestimating the prevalence of these risk factors and related PAF.

The prevalence of underweight as another risk factor of TB was 1.48%. Although underweight is a significant risk factor for TB because of its low prevalence, its related PAF was low (1.47%). In the Chinese study, the PAF for the underweight (BMI<18.5) was 5.52%, which was more than in our study, a reason is a higher prevalence of underweight in china than our study (10.62% vs. 1.48%). PAF depends on association measures and the prevalence of risk factors [14].

Poor nutrition is a significant risk factor for TB [25]. This study estimated the prevalence of the lack of consumption of red meat, chicken, fish, and legumes in the last two weeks. Based on our results, 50.79%, 21.20%, 7.85%, and 5.58% of participants had not consumed fish, red meat, chicken, and legumes in the last two weeks. Our results indicate a considerable poor nutrition status in our study population. We could not calculate the PAF for lack of consumption of the mentioned foodstuffs because we have no access to a measure of association between these risk factors and TB. The protein-energy malnutrition and shortages of micronutrients lead to immunodeficiency, so the host’s susceptibility to infection and active TB will be increased [25, 26]. Based on our results, 20.07% of participants were in the lowest level of SES. The nutrition status of people is associated with SES. There is an inverse association between SES and poor nutrition. People with lower incomes pay less for their diet [27], so a lack of attention to the populations with poor nutrition may lead to a significant challenge in controlling TB.

In this study, we faced some limitations. First, we evaluated the risk factors of TB by self-declaration of participants. This issue increases the risk of information bias because of the restriction in recalling past exposures and people’s reluctance to report sensitive matters such as alcohol consumption, drug use, and jail history. This limitation may lead to underestimating the prevalence of risk factors and related PAF. Second, we could not calculate the PAF for poor nutrition, drug use, working in a wet or dark place, use of fossil fuels, corticosteroid use history, lack of sufficient sunlight in the house, lack of physical activity, and jail history. We had no access to a measure of association between the mentioned risk factors and TB.

Third, because we do not know the prevalence of exposure among TB patients, we could not calculate the PAF based on the adjusted measures of associations such as adjusted RR, OR, and HR by applying the Miettinen formula [28]. Therefore, we used the Levin formula for calculating the PAF based on the crude measure of associations. The calculated PAF by the Levin formula is unbiased in the absence of confounding and effect modification [29]; on the other hand, the observational studies are prone to confounding, and the extracted crude measure of associations may be affected by confounding. Therefore, the estimated PAF for modifiable risk factors of TB in our study should be interpreted with caution.

## Conclusion

Based on this study’s results, poor nutrition, including lack of red meat, chicken, fish, and legumes in the last two weeks, secondhand smoking, smoking, Lack of BCG vaccination, and diabetes were the main modifiable risk factors for TB. The highest PAF for TB were related to secondhand smoke, smoking, lack of BCG vaccination, and diabetes.

## Supporting information

S1 File Supplementary tables. (DOCX)

S2 File Developed questionnaire in Persian. (DOCX)

S3 File Developed questionnaire in English. (DOCX)

S1 Fig A flow chart depicting the stages of retrieving articles and checking eligibility criteria for systematic review. (JPG)
