# Supplementary material for: Prevalence of modifiable risk factors of tuberculosis and their population attributable fraction in Iran: A cross-sectional study
Source: PLoS One. 2022 Aug 4;17(8):e0271511. doi: 10.1371/journal.pone.0271511 (PMC9352083; doi:10.1371/journal.pone.0271511)
Supplement: S1 File — (DOCX) [file pone.0271511.s001.docx]

**S. table 1: characteristics of included studies**

| Author | Year | Country | Study design | Sample size | Risk factor | Type of measure of association | Measure of association | Lower limit | Upper limit |
| --- | --- | --- | --- | --- | --- | --- | --- | --- | --- |
| Alemu, Y. M.(1) | 2016 | Ethiopia | Case-control | 446 | Alcohol | OR | 2.44 | 1.54 | 3.86 |
| Soh(2) | 2017 | China | prospective cohort | 63257 | Alcohol | HR | 1.45 | 1.11 | 1.9 |
| Cheng(3) | 2020 | China | prospective cohort | 34076 | Alcohol | aHR | 0.941 | 0.66 | 1.34 |
| Tewatia(4) | 2020 | India | Case -control | 184 | Alcohol | OR | 1.89 | 1.13 | 3.16 |
| Davis, A(5) | 2107 | Kazakhstan | Case-control | 1600 | Alcohol | aOR | 1.41 | 1.03 | 1.93 |
| Shimeles, E.(6) | 2019 | Ethiopia | Case-control | 260 | Alcohol | OR | 1.13 | 0.61 | 1.27 |
| Mitku(7) | 2016 | Ethiopia | retrospective studies | 571 | Alcohol | OR | 1.81 | 1.1 | 2.96 |
| Stosic(8) | 2016 | Serbia | retrospective-cohort study | 17 | Alcohol | RR | 0.59 | 0.17 | 2.06 |
| Hajarsyah(9) | 2018 | North Sumatera | Case-control | 145 | Lack of BCG scar | OR | 2.03 | 1.44 | 2.87 |
| Siregar(10) | 2019 | Indonesia | Case-control | 43 | Lack of BCG scar | OR | 4.08 | 0.933 | 25.5 |
| Zhang(11) | 2019 | China | cross-sectional | 34269 | Under weight | OR | 1.91 | 1.35 | 2.69 |
| Lin(12) | 2018 | Taiwan | population-based cohort studies (NTC Cohort) | 167392 | Under weight | aOR | 2.37 | 1.46 | 3.58 |
| Lin(12) | 2018 | Taiwan | population-based cohort studies (NHIS Cohort) | 167392 | Under weight | aOR | 2.30 | 0.93 | 4.64 |
| Tewatia(4) | 2020 | India | Case-control | 184 | Under weight | OR | 3.73 | 1.72 | 8.08 |
| Irawan(13) | 2017 | Puskesmas Karangkoba | Case-control | 19 | Under weight | OR | 4.4 | 1.32 | 14.35 |
| Stosic(8) | 2016 | Sebria | retrospective-cohort study | 17 | Under weight | RR | 0.87 | 0.26 | 5.07 |
| Cheng(3) | 2020 | China | elderlies without TB | 34076 | Under weight | aHR | 1.39 | 0.97 | 2.00 |
| Tewatia(4) | 2020 | India | Case-control | 184 | Closed contact with a TB patient | OR | 8.07 | 2.57 | 25.31 |
| Alemu, Y. M.(1) | 2016 | Ethiopia | Case-control | 446 | Previous history of TB | OR | 1.85 | 1.15 | 2.96 |
| kou(14) | 2019 | China | Case-control | 169 | Closed contact with a TB patient | OR | 4.91 | 2.15 | 11.25 |
| Jin, Y.(15) | 2018 | China | Case-control | 86 | Closed contact with a TB patient | OR | 11.50 | 2.71 | 48.77 |
| Jin, Y.(15) | 2018 | China | Case-control | 86 | Family history of TB | OR | 4 | 1.13 | 14.17 |
| Stosic (8) | 2016 | Sebria | retrospective-cohort study | 17 | Family history of TB | RR | 9.28 | 2.99 | 28.79 |
| Alemu, Y. M.(1) | 2016 | Ethiopia | Case-control | 446 | Family history of TB | OR | 1.91 | 1.11 | 3.57 |
| Watanabe(16) | 2016 | Japan | Case-control | 7755 | Previous history of TB | OR | 3.61 | 1.22 | 10.7 |
| Zhang(11) | 2019 | China | cross-sectional | 34269 | Closed contact with a TB patient | OR | 5.90 | 1.85 | 18.79 |
| Malacarne, J.(17) | 2018 | Brazil | Case-control | 153 | Closed contact with a TB patient | OR | 1.5 | 1 | 2.3 |
| Tewatia(4) | 2020 | India | Case-control | 184 | Family history of TB | OR | 9.05 | 2.48 | 32.95 |
| Lee(18) | 2018 | Taiwan | systematic review | 13 | Diabetes | RR | 2.03 | 1.62 | 2.55 |
| Cheng(3) | 2020 | China | prospective cohort | 34076 | Diabetes | HR | 0.62 | 0.32 | 1.21 |
| Zhang(11) | 2019 | China | cross-sectional | 34269 | Diabetes | OR | 1.20 | 0.72 | 2.01 |
| Watanabe(16) | 2016 | Japan | Case-control | 7755 | Diabetes | OR | 4.74 | 1.93 | 11.67 |
| Lin(12) | 2018 | Taiwan | population-based cohort studies(NHIS Cohort) | 167392 | Diabetes | aOR | 1.23 | .74 | 1.97 |
| Lin(12) | 2018 | Taiwan | population-based cohort studies(NTC Cohort) | 167392 | Diabetes | aOR | 1.62 | 1.23 | 2.17 |
| Davis, A(5) | 2107 | Kazakhstan | Case-control(model2) | 1600 | Opium use | aOR | 23.91 | 0.75 | 761.05 |
| Davis, A(5) | 2107 | Kazakhstan | Case-control(model3) | 1600 | Opium use | aOR | 30.98 | 1.67 | 579.68 |
| Soh(2) | 2017 | China | prospective cohort | 63257 | Smoking | aHR | 2.07 | 1.8 | 2.39 |
| Irawan(13) | 2017 | Puskesmas Karangkoba | Case-control |  | Smoking | OR | 3.7 | 1.15 | 11.9 |
| Stosic(8) | 2016 | Sebria | retrospective-cohort study | 17 | Smoking | RR | 0.47 | 0.11 | 2.07 |
| Stosic(8) | 2016 | Sebria | retrospective-cohort study | 17 | Secondhand smoke | RR | 2.71 | 1.01 | 7.28 |
| Alemu, Y. M.(1) | 2016 | Ethiopia | Case-control | 446 | Smoking | OR | 4.73 | 2.3 | 9.72 |
| Siregar(10) | 2019 | Indonesia | Case-control | 43 | Secondhand smoke | OR | 2.61 | 1.08 | 6.28 |
| kou(14) | 2019 | China | Case-control | 169 | Smoking | OR | 2.17 | 1.25 | 3.77 |
| Jin, Y.(15) | 2018 | China | Case-control | 86 | Smoking | OR | 1.55 | 0.67 | 3.56 |
| Malacarne, J.(17) | 2018 | Brazil | Case-control | 153 | Smoking | OR | 5.6 | 2.7 | 11.6 |
| Soh(2) | 2017 | China | prospective cohort | 63257 | Smoking | aHR | 2.07 | 1.80 | 2.39 |
| Shimeles, E.(6) | 2019 | Ethiopia | Case-control | 260 | Smoking | OR | 3.14 | 1.69 | 5.83 |
| Cheng(3) | 2020 | China | elderlies without TB | 34076 | Smoking | HR | 2.27 | 1.36 | 3.78 |
| Watanabe(16) | 2016 | Japan | Case-control | 7755 | Smoking | OR | 0.65 | 0.15 | 2.82 |
| OR: odds ratio; aOR: adjusted odds ratio; RR: relative risk; aRR: adjusted relative risk; HR: hazard ratio; aHR: adjusted hazard ratio | | | | | | | | | |

S. table 2: the estimated measures of association of modifiable risk factors if TB

| Risk factors | Crude OR (95% CI) | Crude RR (95% CI) | Crude HR (95% CI) |
| --- | --- | --- | --- |
| Alcohol consumption | 1.67 (1.05, 2.28)* | 0.59 (0.17, 2.06)** | 1.45 (1.11, 1.90)** |
| Lack of BCG | 2.04 (1.32, 2.75)* | - | - |
| Underweight | 2.01 (1.36, 2.66)* | 0.87 (0.26, 5.07) | - |
| Closed contact with TB patient | 2.83 (0.49, 5.17)* | - | - |
| Family history of TB | 2.03 (0.82, 3.23)* | 9.28 (2.99, 28.79)** |  |
| Diabetes | 1.26 (0.62, 1.90)* | 2.03 (1.62, 2.55)*** | 0.62 (0.32, 1.21)** |
| Smoking | 1.92 (1.23, 2.62)* | 0.47 (0.11, 2.07)** | 2.27 (1.36, 3.78)** |
| Secondhand smoking | 2.61 (1.08, 6.28)** | 2.71 (1.01, 7.28)** |  |

*pooled estimate; **estimate from one study; ***results of a systematic review

Reference

1. Alemu YM, Awoke W, Wilder-Smith A. Determinants for tuberculosis in HIV-infected adults in Northwest Ethiopia: a multicentre case-control study. BMJ open. 2016;6(4):e009058.

2. Soh AZ, Chee CBE, Wang YT, Yuan JM, Koh WP. Alcohol drinking and cigarette smoking in relation to risk of active tuberculosis: prospective cohort study. BMJ open respiratory research. 2017;4(1):e000247.

3. Cheng J, Sun YN, Zhang CY, Yu YL, Tang LH, Peng H, et al. Incidence and risk factors of tuberculosis among the elderly population in China: a prospective cohort study. Infect Dis Poverty. 2020;9(1):13.

4. Tewatia P, Kaushik RM, Kaushik R, Kumar S. Tobacco smoking as a risk factor for tuberculous pleural effusion: a case-control study. Global health, epidemiology and genomics. 2020;5:e1.

5. Davis A, Terlikbayeva A, Aifah A, Hermosilla S, Zhumadilov Z, Berikova E, et al. Risks for tuberculosis in Kazakhstan: implications for prevention. Int J Tuberc Lung Dis. 2017;21(1):86-92.

6. Shimeles E, Enquselassie F, Aseffa A, Tilahun M, Mekonen A, Wondimagegn G, et al. Risk factors for tuberculosis: A case-control study in Addis Ababa, Ethiopia. PloS one. 2019;14(4):e0214235.

7. Mitku AA, Dessie ZG, Muluneh EK, Workie DL. Prevalence and associated factors of TB/HIV co-infection among HIV Infected patients in Amhara region, Ethiopia. African health sciences. 2016;16(2):588-95.

8. Stosic MB, Plavsa D, Mavroeidi N, Jovanovic D, Vucinic V, Stevanovic G, et al. Tuberculosis outbreak among high school students in Novi Pazar, Serbia 2016: a retrospective-cohort study. Journal of infection in developing countries. 2019;13(2):101-10.

9. Hajarsjah N, Daulay RM, Ramayani OR, Dalimunthe W, Daulay RS, Meirina F. Tuberculosis risk factors in children with smear-positive tuberculosis adult as household contact. Paediatrica Indonesiana. 2018;58(2):66-70.

10. Siregar FA, Andayani LS, Sinaga MM. Identification of tuberculosis infection and sociodemographic risk among children who come into household contact with tuberculosis in Medan, Indonesia. Journal of Public Health in Africa. 2019;10(s1).

11. Zhang CY, Zhao F, Xia YY, Yu YL, Shen X, Lu W, et al. Prevalence and risk factors of active pulmonary tuberculosis among elderly people in China: a population based cross-sectional study. Infect Dis Poverty. 2019;8(1):7.

12. Lin HH, Wu CY, Wang CH, Fu H, Lönnroth K, Chang YC, et al. Association of Obesity, Diabetes, and Risk of Tuberculosis: Two Population-Based Cohorts. Clin Infect Dis. 2018;66(5):699-705.

13. Irawan GC, Margawati A, Rosidi A. Underweight increases the risk of pulmonary tuberculosis in adult. Universa Medicina. 2017;36(1):4-10.

14. Kou T, Wang Q, Lv W, Wei B, Liu Y, Zhao S, et al. Poor Sleep Quality Is Associated with a Higher Risk of Pulmonary tuberculosis in Patients with a Type 2 Diabetes Mellitus Course for More than 5 Years. Japanese journal of infectious diseases. 2019;72(4):243-9.

15. Jin Y, Fan JG, Pang J, Wen K, Zhang PY, Wang HQ, et al. Risk of Active Pulmonary Tuberculosis among Patients with Coal Workers'Pneumoconiosis: A Case-control Study in China. Biomedical and environmental sciences : BES. 2018;31(6):448-53.

16. Watanabe A, Matsumoto T, Igari H, Sawa J, Yamaguchi Y, Sakatani M. Risk of developing active tuberculosis in rheumatoid arthritis patients on adalimumab in Japan. Int J Tuberc Lung Dis. 2016;20(1):101-8.

17. Malacarne J, Kolte IV, Freitas LP, Orellana JDY, Souza MLP, Souza-Santos R, et al. Factors associated with TB in an indigenous population in Brazil: the effect of a cash transfer program. Revista do Instituto de Medicina Tropical de Sao Paulo. 2018;60:e63.

18. Lee PH, Fu H, Lee MR, Magee M, Lin HH. Tuberculosis and diabetes in low and moderate tuberculosis incidence countries. Int J Tuberc Lung Dis. 2018;22(1):7-16.
