# Supplementary material for: Prevalence of modifiable risk factors of tuberculosis and their population attributable fraction in Iran: A cross-sectional study
Source: PLoS One. 2022 Aug 4;17(8):e0271511. doi: 10.1371/journal.pone.0271511 (PMC9352083; doi:10.1371/journal.pone.0271511)
Supplement: S2 File — (DOCX) [file pone.0271511.s002.docx]

**بسمه تعالی**

کد پرسشنامه: ................

**با سلام و احترام**

**هموطن گرامی از اینکه وقت ارزشمند خود را در اختیار ما قرار می­دهید بسیار سپاسگزاریم. هدف از این پرسشگری تعیین شیوع عوامل خطر مستعد کننده ابتلا به بیماری سل در استان مرکزی می­باشد. نتایج این بررسی می­تواند به شناسایی افراد در معرض خطر ابتلا به بیماری و همچنین پیشگیری از این بیماری در سطح جامعه به کار گرفته شود. در این پرسشنامه نیازی به ثبت مشخصات فردی خود مثل نام و نام خانوادگی نمی­باشد و اطلاعاتی که ثبت خواهد شد به صورت کاملا محرمانه نزد محققین خواهد بود و اطلاعات فردی در اختیار هیچ فرد حقیقی و یا حقوقی قرار نخواهد گرفت. لذا خواهشمند است در صورت رضایت و تمایل به سوالات این پرسشنامه با دقت پاسخ دهید.**

**امضاء**

**مشخصات پایه:**

سن: ............... جنس: مرد 🞎 زن🞎

محل سکونت:................

شغل:.................

ملیت: ایرانی 🞎 غیر ایرانی🞎 نام ببرید ............

سطح تحصیلات:

بی­سواد🞎 ابتدایی 🞎 راهنمایی🞎

متوسطه/ دیپلم🞎 فوق دیپلم 🞎 لیسانس🞎 فوق لیسانس و بالاتر 🞎

وضیعت تاهل: مجرد🞎 متاهل🞎

وزن: ............................... قد: ..................................

تعداد اعضای خانوار: .......................... مساحت منزل مسکونی به متر ........................

تعداد اتاق منزل مسکونی: .................

**سابقه بیماری­های زمینه­ای:**

1. در حال حاضر به کدامیک از بیماری­های زیر مبتلا هستید؟ (منظور بیماری است که با تشخیص پزشک تحت درمان و یا مراقبت هستید)

دیابت: بلی 🞎 خیر🞎

پرفشاری خون: بلی 🞎 خیر🞎

بیماری کلیوی: بلی 🞎 خیر🞎

بیماری مزمن ریوی: بلی 🞎 خیر🞎

سایر: بلی 🞎 خیر🞎 اگر بلی در صورت تمایل نام ببرید ...................................

1. آیا شما سابقه دریافت عضو (پیوند عضو) داشته اید؟ بلی 🞎 خیر🞎

اگر بلی آیا شما تحت درمان با داروهای ضد رفع پیوند بوده اید؟ نام دارو ...........................

**سوالات مربوط به آگاهی در مورد بیماری سل:**

1. آیا تاکنون در مورد بیماری سل چیزی شنیده­اید؟ بلی 🞎 خیر🞎

اگر بلی لطفا به سوالات شماره 4 تا 7 پاسخ دهید.

1. بیماری سل چگونه به انسان منقل می­شود؟

تنفسی🞎 خوراکی 🞎 تزریقی 🞎 نمی­دانم 🞎

1. آیا زندگی با یک فرد مبتلا به سل خطر ابتلا به بیماری را افزایش می­دهد؟

بلی🞎 خیر 🞎 نمی­دانم 🞎

1. آیا بیماری سل یک بیماری قابل درمان است؟

بلی 🞎 خیر 🞎 نمی­دانم 🞎

1. آیا شما خودتان را در معرض خطر ابتلا به بیماری سل می­دانید؟ بلی 🞎 خیر 🞎

**فاکتورهای خطر ابتلاء به سل:**

1. آیا واکسن ب ث ژ دریافت کرده­اید و یا اسکار(جای زخم) بر روی بازوی شما است؟ بلی 🞎 خیر🞎 نامشخص 🞎
2. آیا سابقه تماس با بیمار مبتلا به سل داشته اید؟ بلی 🞎 خیر🞎 نمی­دانم🞎
3. آیا شما تا به حال دچار علایمی همچون سرفه بیش از دو هفته ، تب ،ضعف و بی حالی و ..... شده اید؟

بلی 🞎 خیر🞎

1. آیا شما شغلی مانند قالی بافی و یا سابقه کار کردن در جای تاریک و نمدار را دارید؟

بلی 🞎 خیر🞎

1. آیا شما در محل کار با سوخت­های فسیلی (مثل چوب، زغال ، نفت و گازوئیل و ....) برای گرم کردن و یا پخت و پز مواجه هستید؟ بلی 🞎 خیر🞎 نمی­دانم🞎
2. آیا شما سابقه مصرف طولانی مدت کورتون و یا دارو های سرکوبگر دستگاه ایمنی (مثل بتامتازون، دگزا متازون، کورتون و ....) دارید؟

بلی 🞎 خیر🞎 نمی­دانم🞎

اگر بلی چه مدتی استفاده می کنید/ می­کردید؟ ................

1. در خانه ای که شما زندگی می کنید آیا نور کافی از پنجره ها وارد خانه می شود؟ بلی 🞎 خیر🞎
2. آیا شما در طول هفته ورزش و یا فعالیت بدنی دارید؟ بلی 🞎 خیر 🞎

اگر بلی چند بار در هفته ............. و به مدت چند دقیقه......................

1. در طول دو هفته گذشته غذاهای زیر در رژیم غذایی شما بوده است؟

گوشت قرمز بلی 🞎 خیر 🞎

گوشت مرغ بلی 🞎 خیر 🞎

ماهی بلی 🞎 خیر 🞎

حبوبات (نخود، لوبیا عدس و ..) بلی 🞎 خیر 🞎

1. آیا شما تاکنون سابقه حصور در زندان و یا زندگی در اردوگاه را داشته­اید؟ بلی 🞎 خیر🞎

اگر بلی چه مدت زمانی: ............

1. آیا یکی از اعضای خانواده شما تاکنون سابقه زندان داشته است؟ بلی 🞎 خیر🞎

اگر بلی چه مدت زمانی: .......................

1. آیا شما سیگار مصرف می­کنید؟ بلی 🞎 خیر🞎

اگر بلی چند نخ در روز: ..................

1. آیا کسی از اعضای خانواده شما (اعضایی که در یک خانه زندگی می­کنید) سیگار می­کشد؟ بلی 🞎 خیر🞎

اگر بلی چند نخ در روز: ..................

1. آیا شما در طول دو هفته گذشته الکل مصرف کرده اید؟ بلی 🞎 خیر 🞎
2. آیا شما در دو هفته گذشته مواد مخدر مصرف کرده اید؟ بلی 🞎 خیر 🞎

اگر بلی نوع مواد مصرفی............

**سوالات مربوط به وضعیت اقتصادی:**

1. آیا شما اتومبیل شخصی (خودرویی که برای کسب درآمد نباشد) دارید؟ بلی 🞎 خیر🞎
2. آیا شما رایانه شخصی (لب تاپ/ رایانه) دارید؟ بلی 🞎 خیر🞎
3. آیا تلفن همراه شما هوشمند است؟ بلی 🞎 خیر🞎
4. آیا شما از اینترنت استفاده می­کنید؟ بلی 🞎 خیر🞎
5. آیا شما در منزل بخچال فریزر ساید یای ساید دارید؟ بلی 🞎 خیر🞎
6. آیا شما در منزل ماشین ظرفشویی دارید؟ بلی 🞎 خیر🞎
7. آیا شما در منزل ماشین لباسشویی دارید؟ بلی 🞎 خیر🞎
8. آیا شما در منزل جارو برقی دارید؟ بلی 🞎 خیر🞎
9. آیا شما در منزل مایکرو ویو دارید؟ بلی 🞎 خیر🞎
10. آیا شما در منزل تلوزیون (ال سی دی یا ال ای دی) LCD/LED دارید؟ بلی 🞎 خیر🞎
