# Supplementary material for: Prevalence of modifiable risk factors of tuberculosis and their population attributable fraction in Iran: A cross-sectional study
Source: PLoS One. 2022 Aug 4;17(8):e0271511. doi: 10.1371/journal.pone.0271511 (PMC9352083; doi:10.1371/journal.pone.0271511)
Supplement: S3 File — (DOCX) [file pone.0271511.s003.docx]

**In the name of God**

ID: ………

**Greetings and Regards**

Dear compatriot, Thank you very much for your valuable time. The purpose of this questionnaire is to determine the prevalence of risk factors for tuberculosis in Markazi province. The results of this study can be used to identify people at risk of disease and also to prevent this disease at the community level. In this questionnaire, there is no need to register your personal details such as name and surname, and the information that will be recorded will be completely confidential to researchers and personal information will not be provided to any natural or legal person. Therefore, if you are satisfied and willing, please answer the questions of this questionnaire carefully.

**Signature**

**Basic characteristics**

**Age: …… Gender:** Male 󠆴󠆴 Female󠆴󠆴

**City of residence: .......**

**Job: ....**

**Nationality:** Iranian 󠆴󠆴 Non Iranian 🞎

**Education:** Illiterate 🞎 Primary: 🞎 Intermediate: 🞎 Diploma: 🞎

Associated degree: 🞎 Bachelor's degree 🞎 Master's degree and higher 🞎

Marital status: Single🞎 Married 🞎

**Wight: ……… Height: ……….**

**Number of household members: .......................... Area of residential house in meters ...............**

**Number of rooms in a residential house: .................**

**History of underlying diseases:**

1. **Which of the following diseases do you currently suffer from? (Refers to a disease that you are being treated or cared for by a doctor)**

Diabetes: Yes 🞎 No 🞎

Hypertension: Yes 🞎 No 🞎

Kidney Disease: Yes 🞎 No 🞎

Chronic lung disease: Yes 🞎 No 🞎

Other: Yes 🞎 No 🞎 If yes, name it if you wish ………

1. **Did you have a history of receiving a member (member link)?**

Yes 🞎 No 🞎

**If yes, have you been treated with anti-transplant drugs? medicine name ...........................**

**Questions about TB awareness:**

1. Have you ever heard of tuberculosis? Yes 🞎 No 🞎

If yes, please answer questions 4 to 7.

1. How is tuberculosis transmitted to humans?

Respiratory 🞎 Oral 🞎 Injectable 🞎 Do not know 🞎

1. Does living with a person with TB increase the risk of getting the disease?

Yes 🞎 No 🞎 Do not know 🞎

1. Is TB a treatable disease?

Yes 🞎 No 🞎 Do not know 🞎

1. Do you consider yourself at risk for TB?

Yes 🞎 No 🞎

**Risk factors for tuberculosis:**

1. Have you received the BCG vaccine or is there a scar (scar) on your arm?

Yes 🞎 No 🞎 Unknown 🞎

1. Have you had contact with a patient with tuberculosis?

Yes 🞎 No 🞎 Do not know 🞎

1. Have you ever had symptoms such as cough for more than two weeks, fever, weakness and lethargy, etc.?

Yes 🞎 No 🞎

1. Do you have a job such as carpet weaving or work experience in a dark and damp place?

Yes 🞎 No 🞎

1. Do you encounter fossil fuels (such as wood, coal, oil and diesel, etc.) for heating or cooking at work?

Yes 🞎 No 🞎 Do not know 🞎

1. Do you have a history of long-term use of corticosteroids or immunosuppressive drugs (such as betamethasone, dexamethasone, corticosteroids, etc.)?

Yes 🞎 No 🞎 Do not know 🞎

If yes, how long have you been using it? ................

1. In the house where you live, does enough light enter through the windows?

Yes 🞎 No 🞎

1. Do you exercise during the week? Yes 🞎 No 🞎

If yes, several times a week ............. and for a few minutes ......................

1. Have you had the following foods in your diet over the past two weeks?

Red meat Yes 🞎 No 🞎

Chicken Yes 🞎 No 🞎

Fish Yes 🞎 No 🞎

Legumes (peas, beans, lentils, etc.) Yes 🞎 No 🞎

1. Have you ever been in prison or living in a camp? Yes 🞎 No 🞎

If yes, how long: ............

1. Has any of your family members ever been in prison? Yes 🞎 No 🞎

If yes, for how long: .......................

1. Do you smoke? Yes 🞎 No 🞎

If yes, a few threads a day: ..................

1. Does anyone in your family (members of the same household) smoke?

Yes 🞎 No 🞎

If yes, a few threads a day: ..................

1. Have you consumed alcohol in the last two weeks? Yes 🞎 No 🞎
2. Have you used drugs in the last two weeks? Yes 🞎 No 🞎

If yes, the type of consumables ............

**Questions related to the economic situation**

1) Do you have a personal car (a car that is not for making money)? Yes 🞎 No 🞎

2) Do you have a personal computer (laptop / computer)? Yes 🞎 No 🞎

3) Is your mobile phone smart? Yes 🞎 No 🞎

4) Do you use the Internet? Yes 🞎 No 🞎

5) Do you have a side-by-side freezer at home? Yes 🞎 No 🞎

6) Do you have a dishwasher at home? Yes 🞎 No 🞎

7) Do you have a washing machine at home? Yes 🞎 No 🞎

8) Do you have a vacuum cleaner at home? Yes 🞎 No 🞎

9) Do you have a microwave at home? Yes 🞎 No 🞎

10) Do you have an LCD / LED TV (LCD or LED) at home? Yes 🞎 No 🞎
