# Supplementary figures and images for: Prevalence of modifiable risk factors of tuberculosis and their population attributable fraction in Iran: A cross-sectional study
Source: PLoS One. 2022 Aug 4;17(8):e0271511. doi: 10.1371/journal.pone.0271511 (PMC9352083; doi:10.1371/journal.pone.0271511)

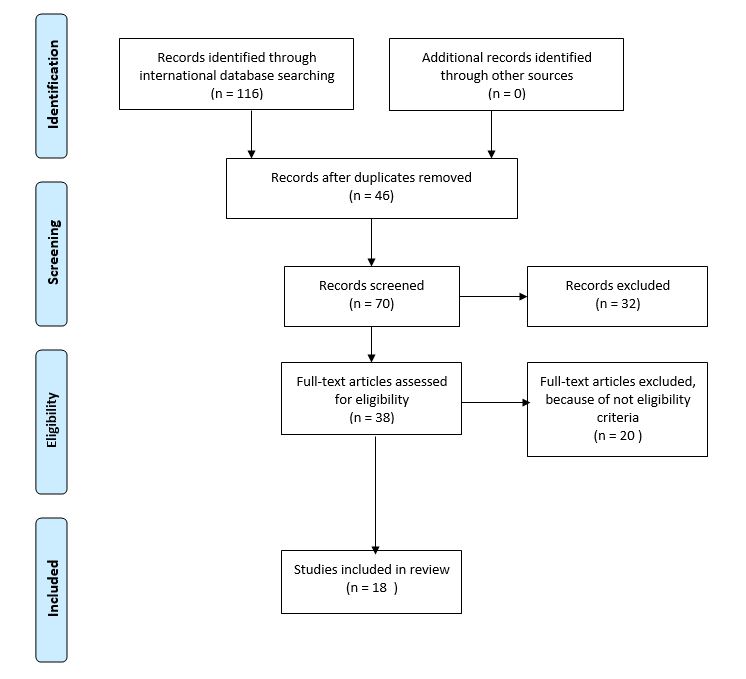

Supplement: S1 Fig — (JPG) [file pone.0271511.s004.JPG]
